# 血液透析成功解救MTHFR C677T基因阳性急性淋巴细胞白血病甲氨蝶呤高敏反应一例

**DOI:** 10.3760/cma.j.issn.0253-2727.2021.06.016

**Published:** 2021-06

**Authors:** 立伟 任, 涛 吴, 锋 薛, 文慧 刘, 东锋 毛, 玲玲 鱼, 宗慧 王, 海 白

**Affiliations:** 解放军联勤保障部队第九四〇医院，兰州 730050 The 940th Hospital of Joint Logistics Support Force of Chinese People's Liberation Army, Lanzhou 730050, China

患者，男，48岁，因“乏力伴心悸、气促2个月余”于2020年2月5日入我科。2020年1月患者无明显诱因出现头晕、乏力、活动后心悸、气促，遂就诊于当地医院。查血常规：WBC 54.52×10^9^/L，淋巴细胞占95.2％，淋巴细胞绝对计数51.93×10^9^/L，HGB 83 g/L，PLT 46×10^9^/L。建议至上级医院进一步就诊。2020年2月5日入院后查骨髓细胞形态学：原始淋巴细胞+幼稚淋巴细胞占92.8％，急性淋巴细胞白血病（ALL）骨髓象。融合基因检测：56种融合基因检测均为阴性。免疫分型：约91.3％的恶性原始幼稚B淋巴细胞，表达CD123、CD9、CD33、CD34、CD10、CD19、HLA-DR，考虑B-ALL。染色体核型检查：未见明显异常。明确诊断为B-ALL BCR-ABL融合基因（−）。3月27日予以VDLP（长春新碱、柔红霉素、左旋门冬酰胺酶、泼尼松）方案化疗，过程顺利。化疗结束后复查骨髓细胞形态学：ALL完全缓解（CR）骨髓象。4月12日予以CAM（环磷酰胺、阿糖胞苷、6-巯基嘌呤）联合方案强化治疗。6月28日入院后复查骨髓细胞形态学：ALL-CR。微小残留病（MRD）检测：CD19^+^淋巴细胞占有核细胞的0.32％，未见明显异常白血病残留细胞（<10^−4^）。7月6日给予大剂量甲氨蝶呤（HD-MTX，3.5 g），化疗数分钟后患者出现烦躁不安、发热、呼吸困难、面色潮红，当即采血查肝肾功能：天冬氨酸转氨酶130 IU/L，丙氨酸转氨酶136 IU/L，尿酸469.0 µmol/L，肌酐141.0 µmol/L，肾小球滤过率50.40 ml·min^−1^·1.73 m^−2^。给予亚叶酸钙（15 mg静脉注射）解救、促排泄治疗后患者症状未见明显缓解。7月7日患者开始出现口腔黏膜溃烂、全身皮肤色素沉着、局部皮肤肿胀。此后给予亚叶酸钙解救（亚叶酸钙15 mg，静脉注射，每日4次，第1～8天）联合口腔黏膜护理、抗感染、升白细胞、输注悬浮红细胞、输注机采血小板、促排泄及对症治疗后患者症状未见明显缓解。7月28日复查肝肾功能：天冬氨酸转氨酶6 IU/L，丙氨酸转氨酶5 IU/L，尿酸363.0 µmol/L，肌酐197.0 µmol/L，肾小球滤过率33.64 ml·min^−1^·1.73 m^−2^。考虑到患者在应用HD-MTX后出现严重的过敏反应，MTX蓄积药物毒性较重，肾功能损害严重，给予亚叶酸钙解救、输血、促排泄、对症支持治疗效果欠佳，与患者家属沟通后，7月30日开始给予连续血液透析治疗，8月18日血液透析结束，复查血常规：WBC 3.34×10^9^/L，HGB 73 g/L，PLT 100×10^9^/L；肝肾功能：天冬氨酸转氨酶15 IU/L，丙氨酸转氨酶14 IU/L，尿酸234.0 µmol/L，肌酐47.0 µmol/L，肾小球滤过率124.96 ml·min^−1^·1.73 m^−2^。患者临床症状明显缓解，肾功能恢复良好，解救成功后出院。患者在治疗过程中出现严重MTX高敏反应，MTX相关高敏基因MTHFR基因SNP位点检测：C677T杂合突变，A1298C正常；SLCO1B1基因位点检测：rs4149056正常。9月14日入院后给予COAD方案维持化疗。10月5日复查骨髓细胞形态学提示CR骨髓象。目前患者仍在后续治疗随访中。

讨论：HD-MTX用于ALL患者维持治疗时引起的相关毒性仍是患者治疗中断或停止的主要原因之一，研究显示，MTHFR C677T基因变异的ALL患者应用MTX更易产生药物蓄积毒性。与其他等位基因相比，MTHFR C677T基因阳性患者在使用HD-MTX诱导治疗第14天时骨髓抑制明显，复发率和死亡率增加。当体内蓄积药物浓度过高时，应用亚叶酸钙解救效果不明显，应用血液透析降低血清药物浓度对改善临床预后具有显著的疗效。

